# Hepatic Artery Infusion Chemotherapy

**DOI:** 10.1155/1990/27090

**Published:** 1990

**Authors:** A. Tuchmann, J. Schüller, A. Kroiss, K. Dinstl

**Affiliations:** 1 1st Dept. of Surgery Rudolfstiftung Vienna Australia; 2 1st Dept. of Internal Medicine Rudolfstiftung Vienna Australia; 3 Institute of Nuclear Medicine Rudolfstiftung Vienna Australia; 4 1st Dept. of Surgery Rudolfstiftung Juchgasse 25 Vienna A-1030 Australia

## Abstract

Hepatic artery chemotherapy was given to 36 patients, using totally implantable devices consisting of a port and external pump. Twenty-seven patients had inoperable liver metastases of colorectal origin. The infusion system was inserted by laparotomy into the hepatic artery via the gastroduodenal artery. There was no operative mortality. Thirteen infusion systems could not be used for chemotherapy due to dislodgement, early death and lack of follow-up. FUdR was infused every two weeks. There were minor local complications like thrombosis of the system and dislodgement of the port. Toxic effects could be managed by reducing the dose. Response to chemotherapy was evaluated by survival, clinical condition, CEA, ultrasound and CT six months after onset of arterial chemotherapy. Ten/twenty-three patients (43%) responded to therapy, eight of them died on the average 19 months after initial chemotherapy. Six patients were non-responders, seven had stable disease. Five/ten patients developed extrahepatic metastases. Mean survival time was 13.1 months, mean interval until relapse 10.6 months.


*HPB Surgery*, 1990, Vol. 2, pp. 21–28
Reprints available directly from the publisher
Photocopying permitted by license only

© 1990 Harwood Academic Publishers GmbH
Printed in the United Kingdom

### HEPATIC ARTERY INFUSION CHEMOTHERAPY

A. TUCHMANN[+], J. SCHÜLLER[++], A. KROISS[+++], and K. DINSTL[+]

*1st Dept. of Surgery[+] (Head: Prof. Dr. K. Dinstl),*
*1st Dept. of Internal Medicine[++] (Head: Prof. Dr. G. Schernthaner),*
*Institute of Nuclear Medicine[+++] (Head: Doz. Dr. A. Kroiss),*
*Rudolfstiftung, Vienna, Austria*

*(Received 29 November 1988; in final form 21 March 1989)*

Hepatic artery chemotherapy was given to 36 patients, using totally implantable devices consisting of a port and external pump. Twenty-seven patients had inoperable liver metastases of colorectal origin. The infusion system was inserted by laparotomy into the hepatic artery via the gastroduodenal artery. There was no operative mortality. Thirteen infusion systems could not be used for chemotherapy due to dislodgement, early death and lack of follow-up. FUdR was infused every two weeks. There were minor local complications like thrombosis of the system and dislodgement of the port. Toxic effects could be managed by reducing the dose. Response to chemotherapy was evaluated by survival, clinical condition, CEA, ultrasound and CT six months after onset of arterial chemotherapy. Ten/twenty-three patients (43%) responded to therapy, eight of them died on the average 19 months after initial chemotherapy. Six patients were non-responders, seven had stable disease. Five/ten patients developed extrahepatic metastases. Mean survival time was 13.1 months, mean interval until relapse 10.6 months.

KEY WORDS: Hepatic metastases, intraarterial chemotherapy, hepatic artery infusion chemotherapy

### INTRODUCTION:

80% of patients who succumb from colorectal cancer die of hepatic metastases[1]. So far hepatic resection has been the only curative intervention: The five year survival rate is 25%[2] and 33%[3]. Nevertheless, the chances of curative resection are no higher than 8%[4].

To help in the incurable state of inoperable liver metastases, palliative methods have been developed:

1. Systemic chemotherapy with a maximum response of 20%[5,6].
2. Regional hepatic chemotherapy has been introduced, presupposing that perfusion of the liver metastases is predominantly by the hepatic artery[7,8]. Regional hepatic artery chemotherapy was first performed via percutaneous angiography catheters[9,10]. However, external catheter-related complications, such as dislodgement, infection or thrombosis were limiting factors with this procedure. Since totally implantable devices with port or pump have been introduced[11,12], this method has become widely accepted. Isolated perfusion of the liver has been performed[13] but is not commonly used. On the other hand, methods to reduce perfusion of hepatic metastases such as hepatic artery

Correspondence: A. Tuchmann, M.D., 1st Dept. of Surgery, Rudolfstiftung, Juchgasse 25, A-1030 Vienna, Austria ligation[14] have been tried, or embolization with microspheres[15]. As an additional measure, microspheres bearing cytostatic drugs have been used[16]. In the absence of palliative procedures, life expectance is as follows: 3.1 months[17], 4 months[18], 11 months[19], and 13.8 months[20].

Being aware of the hopeless condition of these patients, we use regional hepatic chemotherapy. Liver metastases of non-colorectal origin as well as primary tumors of the liver are also included in this series — as has been reported by other authors[21,22,23].

### PATIENTS AND METHODS:

Thirty-six totally implantable infusion systems were inserted between June 1983 and July 1988 (Table 1.) In one patient the catheter system was implanted but metastases could not be confirmed by histological examination. The diagnosis "liver metastases" was assessed by laparotomy (n = 16), ultrasound (n = 10), ultrasound and CT (n = 4), CT (n = 4) and scintigram (n = 1). Fourteen patients received the implantable catheter system at the same time as the resection of the primary tumor and 22 were implanted three to 48 months following primary surgery.

### *Surgical Technique and Infusion Chemotherapy:*

Indications for hepatic artery chemotherapy were hepatic involvement of no more than 70%, expected survival time more than three months and no extrahepatic metastases. The gastroduodenal artery was isolated and the catheter introduced. The distal gastroduodenal artery as well as the right gastric artery and every visible branch were ligated to avoid perfusion of stomach, duodenum and pancreas. The catheter was attached to the port. The latter was fixed in a subcutaneous pouch above the left costal arch. In 18 patients cholecystectomy was carried out, in seven cases this had been performed previously. Liver perfusion was checked postoperatively by radioisotopes ($Xe^{133}$ or $Technetium^{99}$) and angiography (DSA).

Hepatic artery chemotherapy using FUdR was started 10 to 14 days after implantation of the infusion system. 0.3 mg/kg body weight/day FUdR was administered via an external pump (Pharmacia$^{R}$) — two weeks therapy, two-week interval without treatment. These cycles were repeated for six months after which response to therapy was assessed. Response to therapy was defined in terms of a 50% decrease of tumor volume in ultrasound or CT and/or a 30% decrease of CEA levels according to WHO definition. Patients were examined every two weeks. In case of adverse side effects, the dose was reduced to 0.2 or 0.1 mg FUdR/kg/day or the regimen changed to one week of therapy and three week intervals.

**Table 1 Patients**

| | | | |
|---|---|---|---|
| Age | 64 (36–81) years | | |
| Sex | 16 male, | 20 female | |
| Liver metastases | | | 31 |
| colorectal | | 27 | |
| breast | | 2 | |
| uterus | | 1 | |
| choroid membrane | | 1 | |
| Hepatocellular carcinoma | | | 4 |

Patients who did not respond to FUdR or relapsed after an initial response were treated with Mitomycin C 14 mg/m$^2$ body surface, sometimes combined with Spherex$^R$ (microspheres). Hepatocellular carcinomas were treated by Mitoxantrone 14 mg/m$^2$, increasing the dose to 20 mg/m$^2$.

### RESULTS

No patient died after insertion of hepatic artery infusion systems. One system had to be removed because of bowel obstruction following sigmoid resection. One system was not used because of absence of hepatic metastases. In four patients (11%) a dislodgement of the catheter was observed at control angiography, implying that only the left liver lobe (n = 1) or the stomach, pancreas and/or the spleen (n = 3) were perfused. There was one infection of the port (3%), requiring the removal of the system. Two patients did not get chemotherapy because of rapid deterioration: One metastatic liver disease due to breast cancer, one hepatocellular carcinoma in a cirrhotic liver. For three patients who were operated on during the last six months, the time has been too short for evaluation. Two patients stopped chemotherapy within the first three months and they cannot be evaluated. Twenty-three cases remain for evaluation.

*Complications related to the totally implanted system:*
Eight thromboses, four of them treated by streptokinase, four by surgical revision including shortening of the catheter and/or change of the port. All these systems have been working smoothly since these interventions. Two ports had to be revised because of dislodgement.

*Adverse side effects of arterial chemotherapy:*
Pathologic liver function tests were found in more than half of our patients, tests improved rapidly after reducing the dose of FUdR. Only one severe chemical hepatitis was observed. Three patients developed duodenal ulcer, six reported severe gastrointestinal symptoms. There was no cholecystitis and no sclerosing cholangitis in this series. Thirteen patients were free of any side effects during regional chemotherapy.

Response to chemotherapy was evaluated six months after initiation, checking survival, clinical condition, CEA levels, ultrasound and CT (Table 2). Ten/twenty-three (43%) of our patients were considered responders. Nine of these responders belong to the colorectal group, one had liver metastases due to uterine carcinoma. Six were non-responders (Table 2). Four of these patients had a colorectal primary carcinoma, one choroid membrane carcinoma and one a hepatoma. Disease was assessed as stable in seven patients (Table 2). Five of these had metastases due to colorectal cancer, one due to breast cancer and one due to hepatoma. Six/ten responders showed a marked decrease of CEA levels from 343(6–1200) ng/ml before hepatic chemotherapy to 53(3.2–100.8) ng/ml at six months (Table 2). Three/ten patients had a complete remission by ultrasound, 4/10 by CT (Figures 1 and 2). Nevertheless, five out of ten developed extrahepatic metastases although they had responded to therapy at six months: four had lung metastases and one had brain metastasis.

**Table 2** Results

| | *Responder* | *Non-Responder* | *Stable disease* |
|---|---|---|---|
| *n* | *10/23 = 43%* | *6/23* | *7/23* |
| *Survival:* | | | |
| a. alive | 2 after 11 & 27 mos. | 1 after 5 mos. | 6 after 8 (6–10) mos. |
| b. dead | 8 after 19 (12–29) mos. | 5 after 4 (2–7) mos. | 1 after 7 mos. |
| CEA 30% drop | 6/10 | | |
| Ultrasound 50% drop | 3/10 | | |
| CT 50% drop | 4/10 | | |
| Extrahepatic disease | 5/10 | | |
| Mean period between onset of chemotherapy and relapse | | 10.6 (6–16) mos. | |
| Mean survival time | | 13.1 (2–27) mos. | |

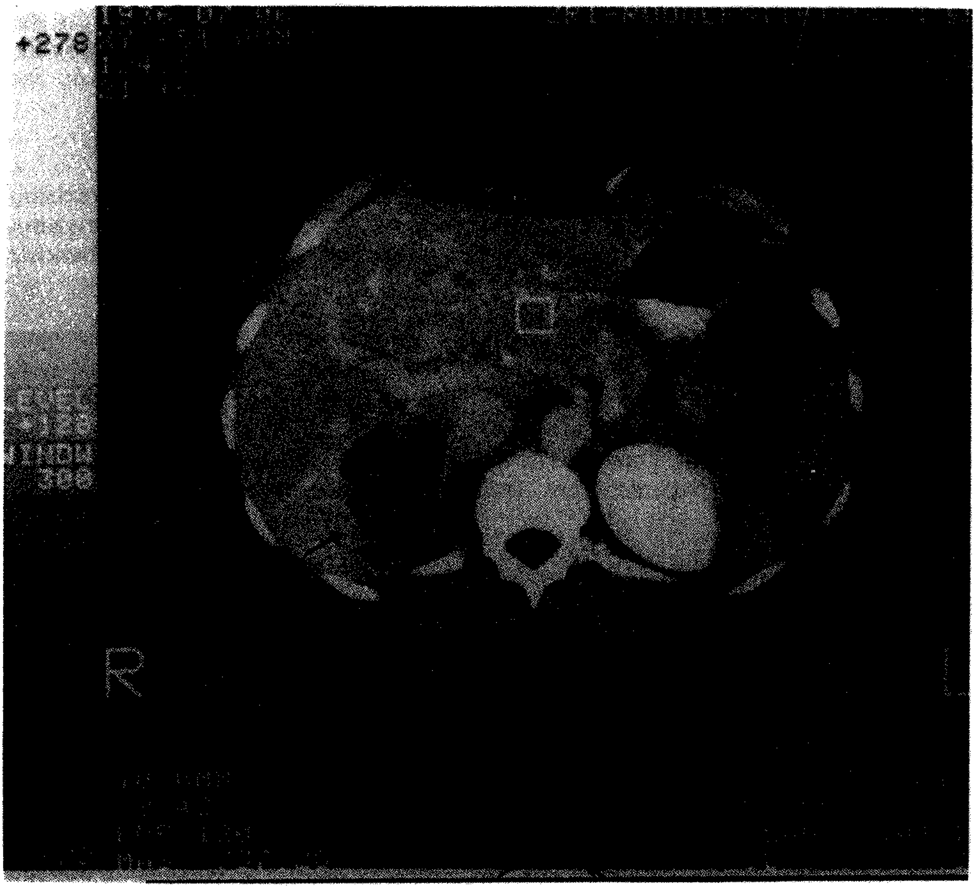

**Figure 1** Hepatic metastases before regional chemotherapy

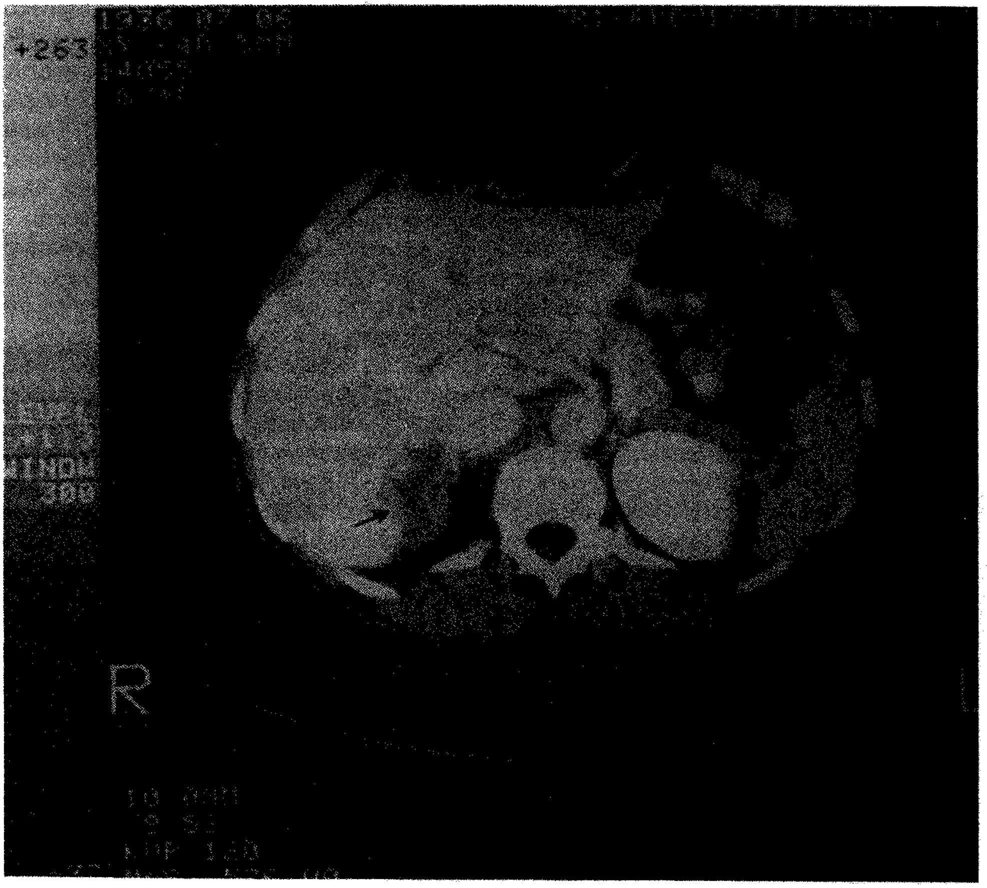

**Figure 2** CT four months after the onset of chemotherapy

### DISCUSSION:

The goals of regional chemotherapy are to maximize concentration of chemotherapeutic drugs. The superiority of hepatic artery chemotherapy compared to the portal vein route was reported in a controlled study[24]. 5-Fluro-2-deoxyuridine (floxuridine, FUdR) is the ideal drug for this purpose because of its high extraction by the liver during the first pass (98%) in comparison to 5-Fluorouracil with a maximum of 50% extraction[25]. Consequently, most authors used FUdR[12,21,26] instead of 5-FU[22,27]. Some[21,28] added Mitomycin C in case of tumor relapse or non-response to FUdR. Discussion of FUdR administration as bolus injection vs. continuous infusion favours continuous administration by pump[11,12,21,28].

Most authors suggest angiography because of possible anomalies of the hepatic artery in 12 – 19%[1,21,29]. The catheter is introduced via the gastroduodenal artery but in appropriate cases the splenic[26] or the gastroepiploic artery (two of our own cases) may be cannulated. Nevertheless, the distal gastroduodenal, the right gastric and also every visible branch supplying stomach, duodenum and pancreas should be ligated. Cholecystectomy to avoid chemical cholecystitis has been performed in every case[30], seldom[28] and occasionally (our own patients). Cohen[28] and our group did not observe subsequent cholecystitis. Nevertheless, we now perform cholecystectomy in every case. Perfusion of the liver may be checked intraoperatively by fluorescein[1,26,30] and postoperatively by radioisotopes (Te[99])[29,20, our own group] or digital subtraction angiography via the port[23, our own group]. In four of our cases the perfusion was misdirected, once to the left liver lobe and in three patients to pancreas and spleen.

The operative mortality is negligible in most reports. There was no postoperative death in our own series. Among our cases there was clotting of the infusion system and dislodgement of the port as well as one infection but local complications do not play an important role in totally implantable infusion systems.

The overall response rate amounted to 43%, comparable with Daly's 41%[24] and Encke's 42%[23]. Niederhuber[12], Balch[21] and Ramming[1] reported much higher response rates ranging between 83[12,21] and 88%[1]. Thirlwell[22] and Chang [30] reported response rates of 60 and 62%, respectively. Nevertheless, response rates cannot be compared as the cases included in the studies were too different: 1. Liver involvement less than 50%[21]; 2. Presence of extrahepatic disease[28,32]; 3. Different chemotherapy regimens and 4. Different criteria of assessment: Survival, clinical condition, sonography, CT and CEA.

Mean survival time could not be markedly prolonged by hepatic artery chemoinfusion: 11.5 to 13 months mean survival in most recent studies[1,23,38, our own cases]. A mean survival time of 24 months [21] could not be observed elsewhere. The mean interval before tumor relapse was seven to nine months[22,23,24,30] compared to 10.6 months in our own patients.

Although there are no systemic side effects of regional chemotherapy[23], the disadvantage of hepatic artery chemotherapy is the toxicity to liver, stomach, duodenum and pancreas. Severe chemical hepatitis was seen in only one instance here, but has constituted 38 to 79% in the literature[23,24,30]. Biliary sclerosis was observed in 15%[23] and 21%[30], gastroduodenal ulcer in 3/23 (13%) of our patients, and 17%[30] or 31%[1] in other series. These toxic side effects depend on the FUdR dose[23,26]; chemical hepatitis is always reversible[21]. The dose always has to be reduced in protracted treatment[23,30, our own experience]. There was no case of biliary sclerosis in our patients, 13/23 subjects had no complaints during FUdR chemoinfusion. We attribute this to an early dose reduction or to a changed cycle consisting of one week therapy and a three week interval. Schlag[27] who worked with 5-FU did not observe any of the adverse side effects described in connection with FUdR.

A major problem of regional hepatic chemotherapy constitutes the developement of extrahepatic tumors, mainly metastatic involvement of the lung. Extrahepatic disease was reported in 45 to 62%[1,23,30] and must be expected in effective chemotherapy of the liver. In our series 5/10 patients with positive response to therapy contracted extrahepatic disease.

Patients have to be selected carefully for this new palliative tumor therapy as a recent prospective randomized study comparing intraarterial with intravenous chemotherapy for liver metastases showed[30]; there were different response rates: 62% (i.a.) vs. 17% (i.v.) but no difference in life expectancy: Two-year survival rate 22 vs. 15%. The disease-free interval was seven and nine months respectively. The goal to be achieved with hepatic artery infusion chemotherapy should be:

1. a less toxic cytostatic agent in order to avoid complications to the bile ducts, the stomach, the duodenum and the pancreas,

2. a combination with systemic chemotherapy to avoid extra-hepatic metastases and
3. a combination with surgery, yielding inoperable metastases operable by regional chemotherapy prior to liver resection.

*References:*

1. Ramming, K.P., O'Toole, K. (1986) The Use of the Implantable Chemoinfusion Pump in the Treatment of Hepatic Metastases of Colorectal Cancer. *Arch. Surg.*, **121**, 1440–1444
2. Adson, M.A., van Heerden, J.A. (1980) Major hepatic resections for metastatic colorectal cancer. *Ann. Surg.*, **191**, 576–583
3. Hughes, K.S., Simon, R., Adson, M.A., *et al.* (1988) Resection of the liver for colorectal carcinoma metastases: A multi-institutional study of indications for resection. Registry of Hepatic Metastases. *Surgery*, **103**, 278–288
4. Morrow, C.E., Grage T.B., Sutherland, D.E.R., *et al.* (1982) Hepatic resections for secondary neoplasms. *Surgery*, **92**, 610–615
5. Moertel, C.G., Reitemeier, R.J., Hahn, R. (1967) A controlled comparison of 5-fluoro-2′-deoxyuridine therapy administered by rapid intravenous injection and by continuous intravenous infusion. *Cancer Res.*, **27**, 549–552
6. Foster, J.H., Lungy, J. (1983) Liver metastases. *Current Probl. Surg.*, **18**, 160–195
7. Breedis, C., Young, G. (1954) The blood supply of neoplasms in the liver. *Am. J. Pathol.*, **30**, 969–985
8. Ackerman, N.B. (1972) Experimental studies on the circulatory dynamics of intrahepatic tumor blood supply. *Cancer*, **29**, 435–441
9. Oberfield, R.A., McCaffrey, J.A., Polio J., *et al.*, (1979) Prolonged and continuous percutaneous intra-arterial hepatic infusion chemotherapy in advanced metastatic liver adenocarcinoma from colorectal primary. *Cancer*, **44**, 413–423
10. Reed, M.A., Vaitkevicius, K., Al-Sarraf, M., *et al.*, (1981) The practicability of chronic hepatic artery infusion therapy of primary and metastatic hepatic malignancies: ten year results of 124 patients in a prospective trial. *Cancer*, **47**, 402–409
11. Buchwald, H., Grage, T.B., Vassilopoulos, P.P., *et al.*, (1980) Intra-arterial infusion chemotherapy for hepatic carcinoma using a totally implantable infusion pump. *Cancer*, **45**, 866–869
12. Niederhuber, J.E., Ensminger, W., Gyves, J., (1984) Regional chemotherapy of colorectal cancer metastatic to the liver. *Cancer*, **53**, 1336–1343
13. Aigner, K., Walther, H., Tonn, J., Links, K.H., Schoch, P., Schwemmle, K., (1984) Die isolierte Leberperfusion bei fortgeschrittenen Metastasen kolorektaler Karzinome. *Onkologie*, **7**, 13–21
14. Balasegaram, M., (1972) Complete Hepatic Dearterialization for Primary Carcinoma of the Liver. *Am. J. Surg.*, **124**, 340–345
15. Lindell, B., Aronsen, K.F., Nosslin, B., Rothman, U., (1978) Studies in pharmacokinetics and tolerance of substances temporarily retained in the liver by microsphere embolization. *Ann. Surg.*, **187**, 95–99
16. Dakhil, S., Ensminger, W., Cho, K., Niederhuber, J., Doan, K., Wheeler, R., (1982) Improved regional selectivity of hepatic arterial BCNU with degradable microspheres. *Cancer*, **50**: 631–635
17. Wood, C.B., Gillis, C.R., Blumgart, L.H., (1976) A retrospective study of the natural history of patients with liver metastases from colorectal cancer. *J. Clin. Oncol.*, **2**, 285–288
18. Jaffe, B.M., Donegan, W.L., Watson, F., Spratt, J.S. (1968) Factors influencing survival in patients with untreated hepatic metastases. *Surg. Gynecol. Obstet.*, **127**, 1–11
19. Wagner, J.S., Adson, M.A., van Heerden, J.A., Adson, M.H., Ilstrup, D.W., (1984) The natural history of hepatic metastases from colorectal cancer. *Ann, Surg.*, **199**, 502–508
20. Cady, B., Monson, D.O., Swinton, N.W., (1970) Survival of patients after colonic resection for carcinoma with simultaneous liver metastases. *Surg Gynecol, Obstet.*, **131**, 697–700
21. Balch, Ch.M., Urist, M.M., McGregor, M.L. (1983) Continuous Regional Chemotherapy for Metastatic Colorectal Cancer Using a Totally Implantable Infusion Pump. *Am. J. Surg.*, **145**, 285–290
22. Thirlwell, M.P., Hollingsworth, L.M., Herba, M.J., Boileau, G., Boos, G., MacFarlane, J.K. (1986) Ambulatory Hepatic Artery Infusion Chemotherapy for Cancer of the Liver. *Am. J. Surg.*, **151**, 585–589
23. Encke, A., Hottenrott, Ch., Lorenz, M., (1987) Die regionale Chemotherapie von Lebermetastasen. Langenbecks *Arch. Chir.*, **371**, 137–148
24. Daly, J.M., Kemeny, N., Sigurdson, E., Oderman, P., Thom, A., (1987) Regional Infusion for Colorectal Hepatic Metastases. A Randomized Trial Comparing the Hepatic Artery with the Portal Vein. *Arch. Surg.*, **122**, 1273–1277

25. Ensminger, W.D., Rosenberg, A., Raso, V., *et al.*, (1978) A clinical-pharmacological evaluation of hepatic arterial infusions of 5-fluoro-2'-deoxyuridine and 5-fluorouracil. *Cancer Res.*, **38**, 3784–3792
26. Daly, J.M., Kemeny, N., Oderman, P., Botet, J., (1984) Long-term Hepatic Arterial Infusion Chemotherapy. Anatomic Considerations, Operative Technique, and Treatment Morbidity. *Arch. Surg.*, **119**, 936–941
27. Schlag, P., Feil, H., Ruoff, G., Hohenberger, P., Hölting, Th., Buhl, K., (1987) Ambulante kontinuierliche intraarterielle Chemotherapie zuhause — ein Erfahrungsbericht. *Schweiz. Med. Wschr.*, **117**, 1342–1346
28. Cohen, A.M., Kaufman, S.D., Wood, W.C., (1985) Treatment of colorectal cancer hepatic metastases by hepatic artery chemotherapy. *Dis. Colon Rectum*, **28**, 389–393
29. Rayner, A.A., Kerlan, R.K., Stagg, R.J., Price, D.C., Hohn D.C., (1986) Total hepatic arterial perfusion after occlusion of variant lobar vessels: Implications for hepatic arterial chemotherapy. *Surgery*, **99**, 708–715
30. Chang, A.E., Schneider, Ph,D., Sugarbaker, P.H., Simpson, C., Culnane, M., Steinberg, S.M., (1987) A Prospective Randomized Trial of Regional Versus Systemic Continuous 5-Fluorodeoxyuridine Chemotherapy in the Treatment of Colorectal Liver Metastases *Ann. Surg.*, **206**, 685–693

*Accepted by S. Bengmark*

### INVITED COMMENTARY

The advent of the technology for hepatic artery infusion using an implantable system with a port has certainly contributed to the management of patients with advanced metastatic diseases of the liver and a considerable number of papers have been published. However, a high incidence rate of complications such as dislodgement of clogging of the catheter, infection around the port, mis-directed perfusion and side effects of anti-tumor agents is a serious problem inherent to this technique. In fact, it is an unacceptable problem, if chemotherapy has to be discontinued because of these complications, especially in the patients in whom the catheter was surgically inserted into the hepatic artery.

In our Department, one-shot transcatheter infusion of an anti-tumor agent suspended in oil (Nimustine-Lipiodol suspension) has been indicated as the method of choice in these cases for the last 3 years and a favorable result has been obtained. Nimustine, a derivative of Nimustine hydrochloride, was suspended in Lipiodol using ultrasonic agitation and used in experimental animals and human subjects. In 2 out of 5 cases with hepatoma which responded to treatment, hepatic lobectomy was carried out some days later. In these cases, although a tiny cancerous lesion was histologically detected at the edge in the subcapsular region a surrounding cystic lesion replaced the tumor, an obvious effect of this agent. In the remainder, administration of anti-tumor agent has been repeated at various intervals when tumor-markers become re-elevated.

Our previous experimental study found that the anti-tumor agent suspended in Lipiodol is more selectively accumulated and more slowly released in tumor tissue, resulting in a long-acting effect, compared with that of an anti-tumor agent such as FUdR dissolved in water. Moreover, another advantage to using Lipiodol is that information necessary for follow-up studies can be easily obtained, because the lesion is clearly delineated with Lipiodol.

The patients in which this type of treatment is indicated are, in general, in a grave condition. Therefore, the type of anti-tumor agent and method of its administration should be seriously considered before it is given.

Tatsuo Yamakawa, M.D., F.A.C.S.
Department of Surgery,
Teikyo University Hospital at Mizonokuchi,
Kawasaki, Japan

